# Antiprotozoal Compounds from *Urolepis hecatantha* (Asteraceae)

**DOI:** 10.1155/2021/6622894

**Published:** 2021-02-11

**Authors:** Orlando G. Elso, Maria Clavin, Natalia Hernandez, Tomás Sgarlata, Hernán Bach, César A. N. Catalan, Elena Aguilera, Guzman Alvarez, Valeria P. Sülsen

**Affiliations:** ^1^Universidad de Buenos Aires, Facultad de Farmacia y Bioquímica, Cátedra de Farmacognosia, Junín 956, Buenos Aires 1113, Argentina; ^2^CONICET–Universidad de Buenos Aires, Instituto de Química y Metabolismo del Fármaco (IQUIMEFA), Junín 956, Buenos Aires 1113, Argentina; ^3^Universidad de Buenos Aires, Facultad de Farmacia y Bioquímica, Museo de Farmacobotánica, Junín 956, Buenos Aires 1113, Argentina; ^4^Instituto de Recursos Biológicos INTA-Hurlingham, De los Reseros y N. Repetto (1686), Hurlingham, Buenos Aires, Argentina; ^5^Universidad Nacional de Tucumán, Facultad de Bioquímica Química y Farmacia, Instituto de Química Orgánica, Ayacucho 471 (T4000INI), San Miguel de Tucumán, Argentina; ^6^Grupo de Química Medicinal-Laboratorio de Química Orgánica, Facultad de Ciencias, Universidad de la República, Montevideo 11400, Uruguay; ^7^Laboratorio de Moléculas Bioactivas, Universidad de la República, CENUR Litoral Norte, Paysandú 60000, Uruguay

## Abstract

The dewaxed dichloromethane extract of *Urolepis hecatantha* and the compounds isolated from it were tested for their *in vitro* activity on *Trypanosoma cruzi* epimastigotes and *Leishmania infantum* promastigotes. The extract of *U. hecatantha* showed activity against both parasites with IC_50_ values of 7 *µ*g/mL and 31 *µ*g/mL, respectively. Fractionation of the dichloromethane extract led to the isolation of euparin, jaceidin, santhemoidin C, and eucannabinolide. The sesquiterpene lactones eucannabinolide and santhemoidin C were active on *T. cruzi* with IC_50_ values of 10 ± 2 *µ*M (4.2 *µ*g/mL) and 18 ± 3 *µ*M (7.6 *µ*g/mL), respectively. Euparin and santhemoidin C were the most active on *L. infantum* with IC_50_ values of 18 ± 4 *µ*M (3.9 *µ*g/mL) and 19 ± 4 *µ*M (8.0 *µ*g/mL), respectively. Eucannabinolide has also shown drug-like pharmacokinetic and toxicity properties.

## 1. Introduction

Chagas' disease and leishmaniasis are protozoan parasitic diseases caused by *Trypanosoma cruzi* and different species of the genus *Leishmania* and transmitted by infected blood-sucking triatomine bugs and phlebotomine sandflies, respectively. They are both classified as neglected tropical diseases by the World Health Organization (WHO) [1].

Chagas' disease or American Trypanosomiasis is a potentially life-threatening disease that affects 6 to 7 million people worldwide. It is estimated that about 30000 new cases occur annually and more than 12000 deaths per year are attributed to this parasitosis [2]. Chagas' disease was historically linked to poor rural areas of Latin America where the insect vector is present. In recent years, the disease has spread to cities and nonendemic areas due to migrations of infected people and nonvectorial transmission of the parasite, turning Chagas' disease into a global public health problem [3]. Nifurtimox and benznidazole are the only drugs currently available for Chagas' disease treatment. Both drugs are effective in the acute stage of the infection and vertical transmission prevention, but their efficacy diminishes in the chronic phase. Besides, frequent adverse events lead to high rates of treatment discontinuation [4]. Therefore, the development of new trypanocidal drugs for Chagas' disease treatment is needed.

Leishmaniasis has three clinical forms: cutaneous, mucocutaneous, and visceral also known as kala-azar. Although cutaneous leishmaniasis is the most common form, visceral leishmaniasis is the most severe form [5]. According to the WHO, more than one billion people are at risk of infection. It is estimated that 30000 new cases of visceral leishmaniasis and more than one million new cases of cutaneous leishmaniasis occur annually [5]. The chemotherapy of leishmaniasis is based on the use of sodium stibogluconate, meglumine antimoniate, pentamidine, amphotericin B, paromomycin, and miltefosine. These drugs are toxic and have other limitations such as the route of administration, length and cost of treatment, and emergence of drug resistance [6].

Natural products play an important role in the drug discovery process. One of the most relevant examples is artemisinin, a sesquiterpene lactone isolated from *Artemisia annua* currently used for malaria treatment [7]. Several natural products with promissory activity against pathogenic protozoa have been reported [8, 9].

*Urolepis hecatantha* (DC) R. M. King and H. Rob. (syn. *Eupatorium hecatanthum* (DC) Baker) is the only species of the monotypic genus *Urolepis* (Asteraceae) [10]. The ethnomedical uses of the aerial parts of *U. hecatantha* by indigenous groups of northeast Argentina have been reported [11–13]. The fresh aerial parts are chewed as antitussive [11], while the infusion or decoction of the aerial parts is used topically for gangrene and ulceration treatment [12]. This species has been employed also as an analgesic for teeth pain treatment [13]. The isolation of flavonoids, terpenoids, and benzofuran derivatives from a collection of *U. hecatantha* from Bolivia has been reported [14].

In this work, we report the isolation of four compounds from *U. hecatantha* from *Argentina* and the evaluation of their *in vitro* activity on *Trypanosoma cruzi* and *Leishmania infantum*. The toxicity and pharmacokinetic properties of the compounds were also estimated.

## 2. Materials and Methods

### 2.1. Plant Materials

The aerial parts of *U. hecatantha* (DC.) R. King and H. Robins (Asteraceae) were collected in Buenos Aires province, Argentina, in March 2018. The plant material was identified and deposited at the Herbarium of the Faculty of Pharmacy and Biochemistry, University of Buenos Aires (BAF 16100).

### 2.2. Extraction and Fractionation Procedures

Grounded dried flowers and leaves of *U. hecatantha* (300 g) were extracted thrice at room temperature with dichloromethane (4.5 L, 6 h). Filtrates were joined and concentrated on a rotary evaporator at 40°C under reduced pressure to give 42 g of crude extract (DE). The crude extract was suspended in ethanol (147 mL) at 60°C, diluted with distilled water (63 mL), and filtered under vacuum. The filtrate was extracted successively with hexane (3 × 60 mL) and dichloromethane (3 × 60 mL). Dichloromethane subextracts were joined and taken to dryness on a rotary evaporator to yield 15 g of dewaxed extract. Dewaxed extract (DDE) was fractionated by silica gel column chromatography (60 × 5 cm, 220 g, 230–400 mesh) and eluted with a gradient of dichloromethane (CH_2_Cl_2_) and increasing amounts of ethyl acetate (EtOAc): 100% CH_2_Cl_2_, CH_2_Cl_2_: EtOAc (9 : 1), (8 : 2), (7 : 3), (6 : 4), (5 : 5), (4 : 6), (3 : 7), (2 : 8), (1 : 9), and 100% EtOAc. Fractions (5 × 200 mL) of each solvent ratio (A_1−5_ to K_1−5_) were collected. All column chromatography fractions were monitored by thin-layer chromatography using silica gel 60 F_254_ plates and anisaldehyde sulphuric as spraying reagent.

### 2.3. Compounds Isolation

Fractions *A*_5_ (100% DCM) and B_1−5_ (DCM: EtOAc 9: 1) were pooled and the solvent was evaporated on a rotary evaporator. The residue obtained was transferred to a small vial with a minimum amount of ethyl acetate and the solution was left at room temperature overnight. From this solution, yellow acicular crystals of compound **1** (euparin, 28 mg) were obtained.

Fractions D_2−5_, eluted with CH_2_Cl_2_ : EtOAc (7 : 3), and the fraction *E*_1_, eluted with CH_2_Cl_2_ : EtOAc (6 : 4), showing a similar profile on TLC, were reunited and brought to dryness on a rotary evaporator. The residue was suspended in a minimum amount of dichloromethane and purified by preparative TLC using silica gel plates. The plates were developed using toluene: EtOAc : formic acid (6 : 4 : 1) as a mobile phase. After drying, plates were analyzed under UV light where a deep green fluorescent band (Rf = 0.6) was observed. The fluorescent band was scraped out and extracted with methanol. After solvent evaporation, a yellow powder (10 mg) identified as jaceidin was obtained.

Fractions H_2-4_, eluted with CH_2_Cl_2_ : EtOAc (3 : 7), were pooled and concentrated under vacuum in a rotary evaporator. The residue was dissolved with a minimum amount of ethyl acetate and allowed to stand at room temperature for 24 hours. From this solution, pure crystals of compound **3** (santhemoidin C, 120 mg) were obtained. Fractions I_3-5_, eluted with CH_2_Cl_2_ : EtOAc (2 : 8), and fractions J_1-3_, eluted with CH_2_Cl_2_ : EtOAc (1 : 9), were combined and brought to dryness on a rotary evaporator. The residue was fractionated by silica gel column chromatography (50 × 3 cm, 150 g, 230–400 mesh) and eluted isocratically with a 2 : 1 mixture of CH_2_Cl_2_ : EtOAc. Twenty fractions of 50 mL each were collected. Fractions 15–17 were reunited and brought to dryness on a rotary evaporator to afford compound **4** (eucannabinolide, 108 mg) as a colourless gum. Both ^1^H- and ^13^C-NMR data of santhemoidin C (**3**) in DMSO-d6 as a solvent are reported here: *δ* 169.9 (C-1”; acetate carbonyl), 169.0 (C-12), 164.5 (C-1′), 150.2 (C-3′), 144.2 (C-4), 136.9 (C-11), 133.5 (C-10), 129.1 (C-1), 125.2 (C-2′), 123.0 (C-5), 120.7 (C-13), 76.6 (C-3), 75.0 (C-6), 72.6 (C-8), 57.7 (C-4′), 57.5 (C-5′), 50.8 (C-7), 42.8 (C-9), 35.4 (C-2), 20.2 (C-2”; acetate methyl), 18.6 (C-14), and 11.8 ppm (C-15); ^1^H-NMR data for santhemoidin C in DMSO-d-6 at 600 MHz: *δ* 4.97 dd (12 and 3.5 Hz; H-1), 2.16 ddd (12, 12, 10; H-2*α*), 2.30 m (H-2*β*), 4.15 ddd (10, 5.5, 4.5; H-3), 4.91 d br (9.8; H-5), 5.08 dd (9.8, 8.5; H-6), 3.22 ddd (8.5, 3.5, 3; H-7), 5.71 m (H-8), 2.64 dd (14.2, 4.4; H-9a), 2.40 dd (14.2, 2; H-9b), 6.14 d (3.5; H-13a), 5.65 d (3.0; H-13b), 1.40 s br (3H; H-14), 1.67 s br (3H; H-15), 6.97 *t* (5.7; H-3′), 4.27 *t* (2H; 5.7; H-4′), 4.76 d (12.1; 5′a), 4.71 d (12.1; 5′b), 1.91 s (3H; C-2”; acetate methyl); Others: O–H at C-3, 5.29 d (4.5); O–H at C-4′, 5.18 *t* (5.7).

See spectra in supplementary material. Assignments were made by H–H COSY, HSQC, and HMBC experiments.

### 2.4. Spectrometric Analyses

The isolated compounds were identified by proton nuclear magnetic resonance (^1^H-NMR) and carbon nuclear magnetic resonance (^13^C-NMR), heteronuclear single quantum correlation (HSQC), heteronuclear multiple bond correlation (HMBC), correlated spectroscopy (COSY) (Bruker Advance 600) (600 MHz in CDCl_3_), and electron impact-mass spectrometry (EI-MS).

The purity of santhemoidin C (**3**), estimated by ^1^H-NMR, was >95%. Likewise, the purity estimated for euparin (**1**) and eucannabinolide (**4**), also by ^1^H-NMR, was 97.5% and *ca*. 94%, respectively (see the corresponding ^1^H-NMR spectra in supplementary material). Jaceidin sample was analyzed by TLC using CH_2_Cl_2_ : EtOAc as a solvent and a 10% solution of antimony (III) chloride in chloroform as spray reagent. A single spot was observed under long-wave UV light. It was identified by its mp 131–135°C (“Jaceidin,” Human Metabolome Database, HMDB0033819) and by chromatographic analysis with an authentic sample and confirmed by UV spectroscopy with shift reagents.

### 2.5. Antiparasitic Activity Assay

For the anti-*Trypanosoma* and anti-*Leishmania* activity assays, we followed the methods described by Aguilera et al., 2019 [15].

For the *in vitro* anti-*Trypanosoma cruzi* activity, epimastigotes of the Tulahuen 2 strain (genotype TcVI) grown in an axenic medium (BHI-Tryptose) were used. Cells from a 5–7-day-old culture were inoculated in a fresh culture medium to give an initial concentration of 10^6^ cells/mL. The absorbance at 600 nm of the cells in culture was measured every day. On day five, the medium was inoculated with different doses of the compounds (25–0.05 *µ*M) from a stock solution in dimethylsulfoxide (DMSO) (DMSO concentration in the culture medium never exceeded 0.4%). Control parasites were cultivated in medium with 0.4% DMSO v/v. Benznidazole was used as a positive control. At five days, the absorbance of the culture was measured and compared to the control and the IC_50_ values were calculated for each compound using OriginLab 8.5® sigmoidal regression. Each experiment was done in duplicate, and each concentration was tested in triplicate.

*Leishmania infantum* (MHOM/BR/2002/LPC-RPV) was obtained from Fiocruz (Collection of Oswaldo Cruz Foundation, Rio de Janeiro, Brazil). Promastigotes were cultured as described [16] with some modifications at 28°C in an axenic medium (BHI-Tryptose supplemented with: FBS 10%, hemine 2 × 10^−5^ mg/mL, glucose 3.0 × 10^−4^ g/mL, streptomycin 2.0 × 10^−4^ g/mL, ampicillin 1.3 × 10^−4^ g/mL) as a low-cost alternative for *Leishmania* spp. culture. Assays were performed in 96-well plastic plates using 2 × 10^6^ promastigotes per well. Compounds were dissolved in DMSO. Different serial dilutions (25–0.05 *µ*M) of the compounds with a final volume up to 200 *µ*L were added. After 48 h at 28°C, 20 *µ*L of a 2 mM resazurin solution was added, and the oxidation-reduction was quantified at 570 and 600 nm. The resazurin solution was prepared at 2.5 mM in phosphate-buffered solution (PBS), pH 7.4, and filtered through 0.22 *µ*m before use. Resazurin sodium salt was obtained from Sigma-Aldrich (St. Louis, MO, USA) and stored at 4°C protected from light. Glucantime was used as a positive control. The efficacy of each compound was estimated by calculating the IC_50_ values using OriginLab 8.5^®^ sigmoidal regression. Each antiproliferative experiment was done in duplicate, and each concentration was tested in triplicate.

### 2.6. Cytotoxicity Assay

The cytotoxicity of the dewaxed dichloromethane extract of *U. hecatantha* and the isolated compounds was evaluated according to the method described by Aguilera et al., 2019 [15]. The J774.1 murine macrophages (ATCC, USA) were grown in a DMEM culture medium containing 4 mM L-glutamine and supplemented with 10% FCS. Cells were seeded in a 96-well plate (5.00 × 10^4^ cells in 200 *µ*L culture medium) and incubated at 37°C in a 5% CO_2_ atmosphere for 48 h, to allow cell adhesion before drug testing. Afterwards, cells were exposed for 48 h to the compounds (25–400 *µ*M) or the vehicle for control (medium with 0.4% DMSO v/v), and additional control (cells in medium) were used in each test. Cell viability was then assessed by measuring the mitochondria-dependent reduction of MTT [3-(4,5-dimethylthiazol-2-yl)-2,5-diphenyltetrazolium bromide] to formazan. For this purpose, MTT in sterile PBS (containing 0.2% glucose), pH 7.4, was added to the macrophages to achieve a final concentration of 0.1 mg/mL, and the cells were incubated at 37°C for 3 h. After removing the medium, formazan crystals were dissolved in 180 *µ*L of DMSO and 20 *µ*L of MTT buffer (0.1 M glycine, 0.1 M NaCl, 0.5 mM EDTA, pH 10.5), and the absorbance at 560 nm was measured. The CC_50_ was defined as the drug concentration at which 50% of the cells were viable, relative to the control (no drug added), and was determined using OriginLab 8.5® sigmoidal regression (% of viable cells compared to the logarithm of the compound concentration). Tests were performed in triplicate.

### 2.7. Toxicity and Pharmacokinetic Properties

The toxicity and pharmacokinetic properties of the compounds were estimated with the open-access SwissADME software (http://www.swissadme.ch), a tool that allows the prediction of different pharmacokinetic parameters such as water solubility, gastrointestinal absorption, skin penetrability, lipophilicity, bioavailability, and so forth and T. E. S. T (Toxicity Estimation Software Tool). The software input uses the SMILES codes of the molecules, which were generated with the ChemBioOffice 2010 program.

### 2.8. Statistical Analysis

The statistical analysis was performed using Origin software package version 7.0. The statistical significance of the difference between the data pairs was evaluated by analysis of variance (one-way ANOVA), followed by the Tukey test. Statistical differences were considered significant at *p* < 0.05.

## 3. Results and Discussion

**Figure 1:**
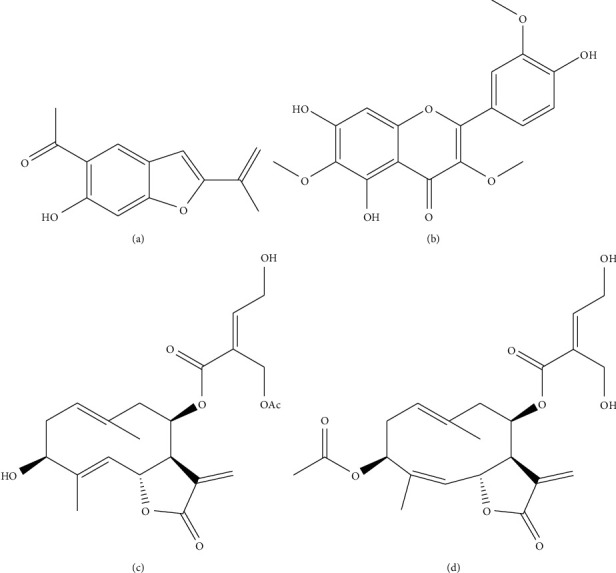
Chemical structures of euparin (a), jaceidin (b), santhemoidin C (c), and eucannabinolide (d).

The dewaxed dichloromethane extract (DDE) of the aerial parts of *U. hecatantha* was evaluated against *T. cruzi* epimastigotes and *L. infantum* promastigotes. This extract was active against *T. cruzi* and *L. infantum* with 50% inhibitory concentration (IC_50_) values of 7 *µ*g/mL and 31 *µ*g/mL, respectively. The *in vitro* cytotoxic effect of the DDE was evaluated on murine macrophages by the MMT method. This extract showed a 50% cytotoxic concentration (CC_50_) value of 15 *µ*g/mL. Fractionation of the DDE by column chromatography and purification of the subfractions by chromatographic techniques yielded four compounds: compound **1** (0.0093%), compound **2** (0.0033%), compound **3** (0.04%), and compound **4** (0.036%). The compounds were identified by spectroscopic methods as euparin (**1**), jaceidin (**2**), santhemoidin C (**3**), and eucannabinolide (**4**) (Figure 1).

Euparin and eucannabinolide have been previously isolated from *U. hecatantha* collected in Bolivia [14]. The presence of santhemoidin C and jaceidin in this species is reported for the first time. The sesquiterpene lactones santhemoidin C and eucannabinolide have been described in *Schkuhria anthemoidea* [17]. Eucannabinolide, jaceidin, and euparin have also been reported in other Asteraceae species [18–21].

**Figure 2:**
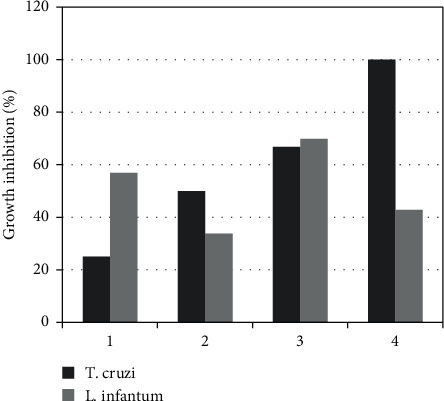
Effect of compounds 1–4 (25 *µ*M) on *T. cruzi* and *L. infantum*.

The *in vitro* antiprotozoal activity of the isolated compounds (**1–4**) was evaluated against *T. cruzi* epimastigotes and *L. infantum* promastigotes (Figure 2). The sesquiterpene lactone eucannabinolide (**4**) was the most active on *T. cruzi* with an IC_50_ value of 10 ± 2 *µ*M (4.2 *µ*g/mL). Santhemoidin C (**3**) displayed also a trypanocidal activity with an IC_50_ of 18 ± 3 *µ*M (7.6 *µ*g/mL). On the other hand, euparin (**1**) and jaceidin (**2**) showed moderate activity against epimastigotes with IC_50_ values > 25 *µ*M. The IC_50_ for the positive control benznidazole was 7 ± 2 *µ*M.

Euparin (**1**) and santhemoidin C (3) were the most active on *L. infantum* with IC_50_ values of 18 ± 4 *µ*M (3.9 *µ*g/mL) and 19 ± 4 *µ*M (8.0 *µ*g/mL), respectively. The flavonoid jaceidin (**2**) and the sesquiterpene lactone eucannabinolide (**4**) were less active (IC_50_ > 25 *µ*M). Glucantime showed an IC_50_ value of 26 ± 9 *µ*M.

The cytotoxic effect of compounds **1**–**4** was assayed on mammalian cells. The terpenoid compounds, santhemoidin C (**3**) and eucannabinolide (**4**), showed CC_50_ values of >15 *µ*M and 15 *µ*M, respectively. Euparin (**1**) and jaceidin (**2**) presented CC_50_ values > 25 *µ*M. Taking in consideration that selectivity is a relevant characteristic for defining hit molecules, selectivity indexes (SI) of the compounds were calculated. The most active compound against *T. cruzi*, eucannabinolide (**4**), showed a SI value of 1.5.

The differences in activity between *T. cruzi* and *L. infantum* for compound **4** compared to compound **3** are remarkable. Both are germacranolides with the same molecular formula (C_22_H_28_O_8_) but differ in the stereochemistry of the C4–C5 double bond: lactone **3** is a germacrolide (a *trans, trans*-germacranolide) while lactone **4** is a heliangolide (a *trans*, *cis*-germacranolide) [22]; they also differ in the location of the acetyl group which in lactone **3** esterifies the hydroxyl group of the 4,5-dihydroxytigloyloxy ester residue at C-8, while, in lactone **4,** it esterifies the hydroxyl group at C-3 of the heliangolide skeleton. These differences should be expected to strongly affect conformation, electronic distribution, and hydrogen bonding interactions. Therefore, these stereochemical and positional changes could increase the activity of compound **4** on *T. cruzi* and decrease it for *L. infantum*. Santhemoidin C, euparin, and jaceidin did not display selectivity on *T. cruzi* epimastigotes. None of the compounds showed selectivity against *L. infantum* promastigotes.

The pharmacokinetic characteristics and toxicity of the compounds play an important role in the drug discovery process. These properties are influenced in part by the physicochemical properties of drugs. In this sense, the mutagenicity, the oral rat LD_50_, the Log P, solubility, gastrointestinal (GI) absorption, skin permeation (Log Kp), and the blood-brain barrier (BBB) permeant were predicted (Table 1). Euparin (**1**), santhemoidin C (**3**), and eucannabinolide (**4**) showed no mutagenicity and LD_50_ values > 600 mg/kg with high GI absorption. Santhemoidin C and eucannabinolide presented log *P* values lower than 2, satisfying the criteria established by Lipinski [23] and showed the best skin permeation. Compounds **2**–**4** showed no BBB permeation.

Eucannabinolide has shown activity against *Trypanosoma brucei rhodesiense* trypomastigotes (IC_50_ = 1.1 ± 0.1 *µ*M) and has tested its cytotoxicity on mammalian cells (L6-cell line from rat-skeletal myoblasts; CC_50_ = 7.8 ± 0.8 *µ*M) [24]. This sesquiterpene lactone was also active when it was loaded onto polylactic acid nanoparticles with a free drug equivalent IC50 value of 3.32 *µ*M [25]. Eucannabinolide has also exhibited cytotoxic activity against tumour cell lines [26] and anti-inflammatory activity [27]. Compound **4** has also shown drug-like pharmacokinetic and toxicity properties (Table 1). No reports about the biological activities of santhemoidin C have been found. Antibacterial and antiviral activity [28, 29] and protective effect on human lymphocytes against chromosomal damage [30] have been reported for jaceidin. Euparin has shown antipoliovirus activity [31] and cytotoxic activity against liver carcinoma cells [32]. This is the first time that the activity against *T. cruzi* and *L. infantum* of these compounds has been reported.

## 4. Conclusions

In this study, the activity against *T. cruzi* and *L. infantum* of the dichloromethane extract of *U. hecatantha* and the isolation of four compounds, euparin, jaceidin, santhemoidin C, and eucannabinolide, are reported. This is the first communication describing the isolation of santhemoidin C and jaceidin from *U. hecatantha*. The activity of the isolated compounds against *T. cruzi* and *L. infantum* is being reported for the first time. The sesquiterpene lactone eucannabinolide was the most active compound against *T. cruzi* and could be considered for further studies.

## Figures and Tables

**Table 1:** Predicted toxicity and pharmacokinetic profile of the isolated compounds.

Compound	Mutagenicity by Ames test	Oral rat LD_50_ (mg/kg)	Consensus Log P o/w	Solubility (mg/mL)	GI absorption	Log kp (skin permeation) cm/s	BBB permeant
1	Negative	620	2.82	3.77e − 02	High	−5.03	Yes
2	Positive	303	2.15	3.42e − 02	High	−6.52	No
3	Negative	680	1.69	1.85	High	−8.45	No
4	Negative	680	1.63	1.85	High	−8.45	No

## Data Availability

The data used to support the findings of this study are included within the article.

## Supplementary Materials

Supplementary Materials The NMR and MS spectra of the compounds were included as supplementary materials.

## References

[1] World Health Organization (WHO) (2020). *Neglected Tropical Diseases*.

[2] World Health Organization (WHO) (2020). *Chagas Disease (American Trypanosomiasis)*.

[3] Lidani K. C. F., Andrade F. A., Bavia L. (2019). Chagas disease: from discovery to a worldwide health problem. *Frontiers in Public Health*.

[4] Pérez-Molina J. A., Crespillo-Andújar C., Bosch-Nicolau P., Molina I. (2020). Trypanocidal treatment of Chagas disease. *Enfermedades Infecciosas Y Microbiología Clínica*.

[5] World Health Organization (WHO) (2020). *Leishmaniasis*.

[6] Bhattacharya A., Corbeil A., do Monte-Neto R. L. (2020). Of drugs and trypanosomatids: new tools and knowledge to reduce bottlenecks in drug discovery. *Genes (Basel)*.

[7] Tu Y. (2016). Artemisinin-A gift from traditional Chinese medicine to the World (nobel lecture). *Angewandte Chemie International Edition*.

[8] Tajuddeen N., Van Heerden F. R. (2019). Antiplasmodial natural products: an update. *Malaria Journal*.

[9] Schmidt T. J., Khalid S. A., Romanha A. J. (2012). The potential of secondary metabolites from plants as drugs or leads against protozoan neglected diseases - part II. *Current Medicinal Chemistry*.

[10] King R. M., Robinson H. (1987). The genera of the eupatorieae (Asteraceae). *Monographs in Systematic Botany*.

[11] Filipov A. (1994). Medicinal plants of the pilagá of central chaco. *Journal of Ethnopharmacology*.

[12] Martínez G. J., Barboza G. E. (2010). Natural pharmacopoeia used in traditional Toba medicine for the treatment of parasitosis and skin disorders (Central Chaco, Argentina). *Journal of Ethnopharmacology*.

[13] Martinez G G. (2010). Natural remedies in the prevention and oral health care of the Toba from Central Chaco (Argentina). *Boletín Latinoamericano y del Caribe de Plantas Medicinales y Aromáticas*.

[14] Gutierrez A., Catalan C., Dias J. (1995). Sesquiterpene lactones, a labdane and other constituents of *Urolepis hecatantha* and *Chromolaena arnottiana*. *Phytochemistry*.

[15] Aguilera E., Perdomo C., Espindola A. (2019). A nature-inspired design yields a new class of steroids against trypanosomatids. *Molecules*.

[16] Faral-Tello P., Greif G., Satragno D., Basmadjián Y., Robello C. (2020). *Leishmania infantum* isolates exhibit high infectivity and reduced susceptibility to amphotericin B. *RSC Medicinal Chemistry*.

[17] Pérez A. L., Mendoza J. S., de Vivar A. R. (1984). Germacranolides from *Schkuhria anthemoidea*. *Phytochemistry*.

[18] Herz W., Govindan S. V. (1980). Eucannabinolide and other constituents of *Schkuhria virgata*. *Phytochemistry*.

[19] Aritomi M., Kawasaki T. (1984). Three highly oxygenated flavone glucuronides in leaves of *Spinacia oleracea*. *Phytochemistry*.

[20] Sanz J. F., Marco J. A., Rustaiyan A. (1990). Chemical constituents from *Centaurea persica* and *Senecio coronopifolius*. *Pharmazie*.

[21] Ghazy N. M., El-Masry S. (1986). Two eremophilanes from *Senecio desfontainei* druce. *Acta Pharmaceutica*.

[22] Sülsen V. P., Catalán C. A. N., Martino V. S., Sülsen V. P., Martino V. S. (2018). Analytical procedures. *Sesquiterpene Lactones. Advances in Their Chemistry and Biological Aspects*.

[23] Lipinski C. A., Lombardo F., Dominy B. W., Feeney P. J. (1997). Experimental and computational approaches to estimate solubility and permeability in drug discovery and development settings. *Advanced Drug Delivery Reviews*.

[24] Kimani N. M., Matasyoh J. C., Kaiser M., Brun R., Schmidt T. J. (2018). Antiprotozoal sesquiterpene lactones and other constituents from tarchonanthus camphoratus and Schkuhria pinnata. *Journal of Natural Products*.

[25] Kimani N., Backhaus S., Matasyoh J. (2019). Preparation of sesquiterpene lactone-loaded PLA nanoparticles and evaluation of their antitrypanosomal activity. *Molecules*.

[26] Woerdenbag H. J., Hendriks H., Malingré T. M., van Stralen R., van den Berg K. J., Konings A. W. T. (1988). In vitro cytotoxicity of sesquiterpene lactones fromEupatorium cannabinum L. and semi-synthetic derivatives from eupatoriopicrin. *Phytotherapy Research*.

[27] Kudumela R. G., Mazimba O., Masoko P. (2019). Isolation and characterisation of sesquiterpene lactones from *Schkuhria pinnata* and their antibacterial and anti-inflammatory activities. *South African Journal of Botany*.

[28] Allison B. J., Allenby M. C., Bryant S. S., Min J. E., Hieromnimon M., Joyner P. M. (2017). Antibacterial activity of fractions from three Chumash medicinal plant extracts and in vitro inhibition of the enzyme enoyl reductase by the flavonoid jaceosidin. *Natural Product Research*.

[29] Elsohly H., El-Feraly F., Joshi A., Walker L. (1997). Antiviral flavonoids fromAlkanna orientalis. *Planta Medica*.

[30] Aljancic I., Stankovic M., Teševic V. (2010). Protective effect on human lymphocytes of some flavonoids isolated from two *Achillea* species. *Natural Product Commununications*.

[31] Visintini Jaime M. F., Campos R., Martino V. (2013). Antipoliovirus activity of the organic extract of *Eupatorium buniifolium*: isolation of euparin as an active compound. *Evidence-based Complementary and Alternative Medicine*.

[32] Ezzat M. I., Ezzat S. M., El Deeb K. S., El Fishawy A. M. (2017). In vitro evaluation of cytotoxic activity of the ethanol extract and isolated compounds from the corms of *Liatris spicata* (L.) willd on HepG2. *Natural Product Research*.

