# Supplementary material for: Antiprotozoal Compounds from Urolepis hecatantha (Asteraceae)
Source: Evid Based Complement Alternat Med. 2021 Feb 11;2021:6622894. doi: 10.1155/2021/6622894 (PMC7895558; doi:10.1155/2021/6622894)
Supplement: Supplementary Materials — The NMR and MS spectra of the compounds were included as supplementary materials. [file 6622894.f1.pdf]

Supplementary Materials

Antiprotozoal compounds from *Urolepis hecatantha* (Asteraceae)

Orlando G. Elso[a,b], Maria Clavin[a,b], Natalia Hernandez[a], Tomás Sgarlata[a], Hernán Bach[c,d], César A. N. Catalan[e], Elena Aguilera[f], Guzman Alvarez[g,†], Valeria P. Sülsen[a,b,*,†]

[a] Universidad de Buenos Aires, Facultad de Farmacia y Bioquímica, Cátedra de Farmacognosia, Junín 956 2° floor (1113). Buenos Aires, Argentina.

[b] CONICET – Universidad de Buenos Aires. Instituto de Química y Metabolismo del Fármaco (IQUIMEFA), Junín 956 2° floor (1113). Buenos Aires, Argentina.

[c] Universidad de Buenos Aires, Facultad de Farmacia y Bioquímica, Museo de Farmacobotánica, Junín 956 1° floor (1113). Buenos Aires, Argentina.

[d] Instituto de Recursos Biológicos INTA-Hurlingham, De los Reseros y N. Repetto (1686) , Hurlingham, Buenos Aires, Argentina

[e] Universidad Nacional de Tucumán. Facultad de Bioquímica Química y Farmacia. Instituto de Química Orgánica. Ayacucho 471 (T4000INI). San Miguel de Tucumán, Argentina.

[f] Grupo de Química Medicinal-Laboratorio de Química Orgánica, Facultad de Ciencias, Universidad de la República, Montevideo, C.P. 11400, Uruguay.

[g] Laboratorio de Moléculas Bioactivas, Universidad de la República, CENUR Litoral Norte, C.P. 60000, Paysandú, Uruguay.

* Corresponding author:

Tel: +54-11-5287-4286; Fax: 54-11-4508-3642

E-mail: vsulsen@ffyb.uba.ar; E-mail: guzmanalvarezlqo@gmail.com

† The work was co-directed by both authors.

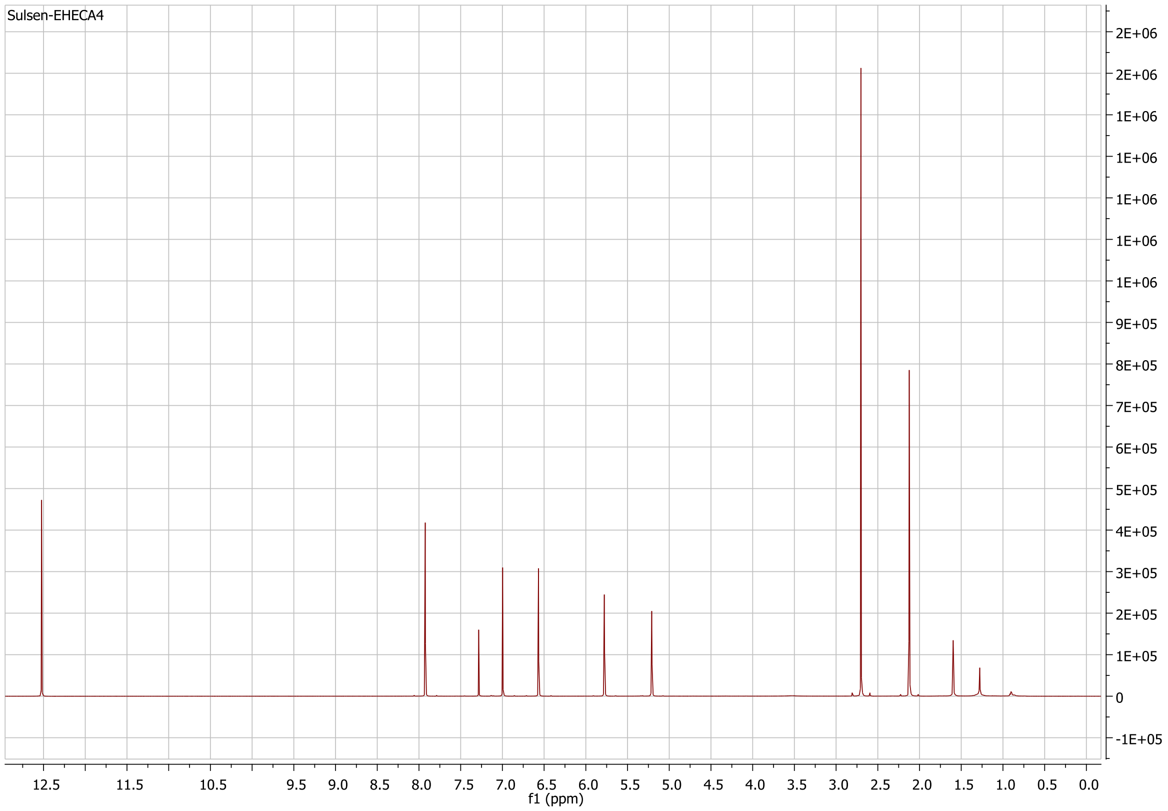

Figure S1. ^{1}H-NMR spectrum of euparin in $CDCl_3$.

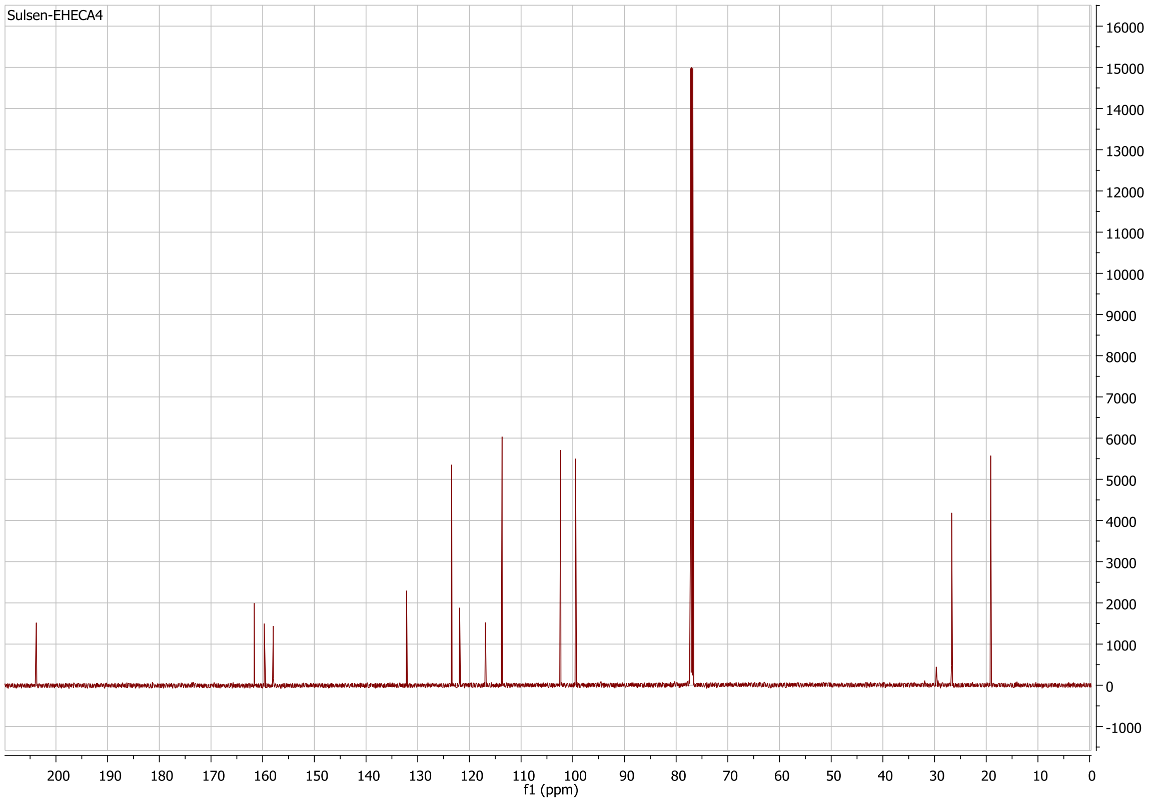

Figure S2. ^{13}C-NMR spectrum of euparin in $CDCl_3$.

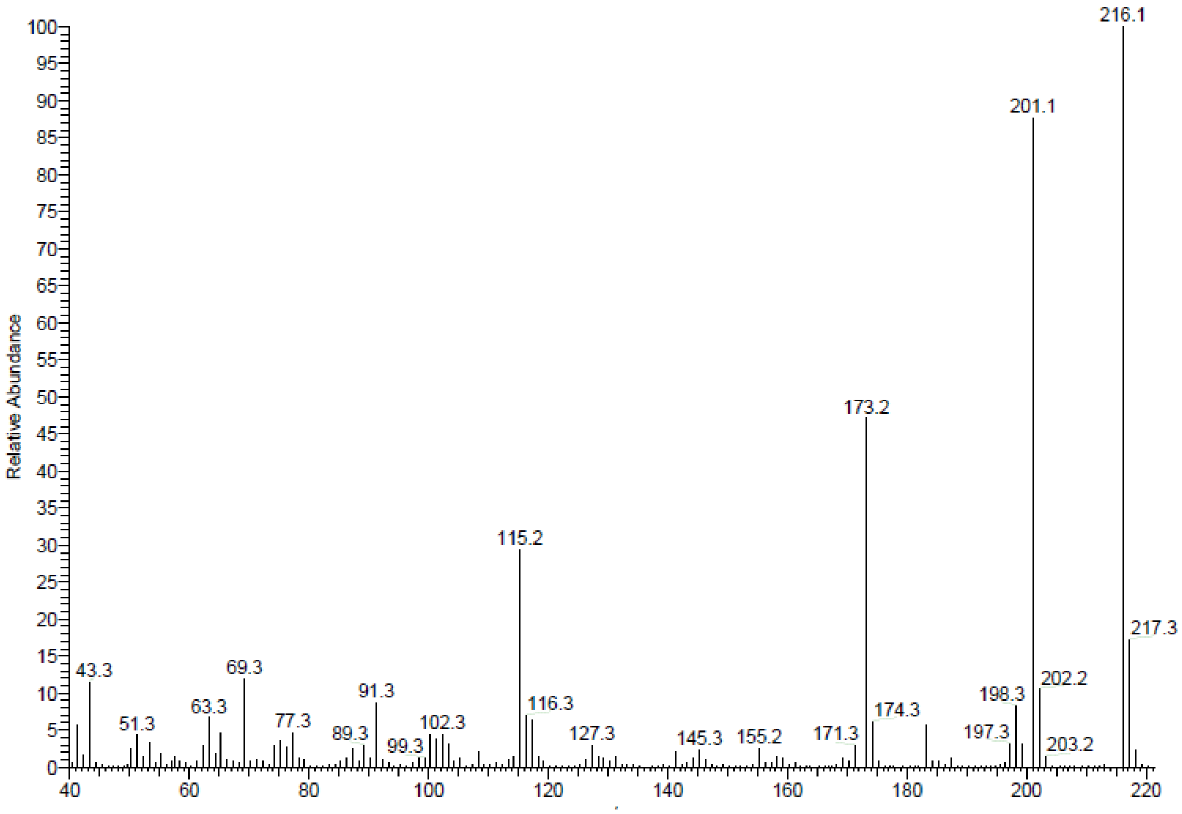

Figure S3. EI-MS spectrum of euparin (70 eV).

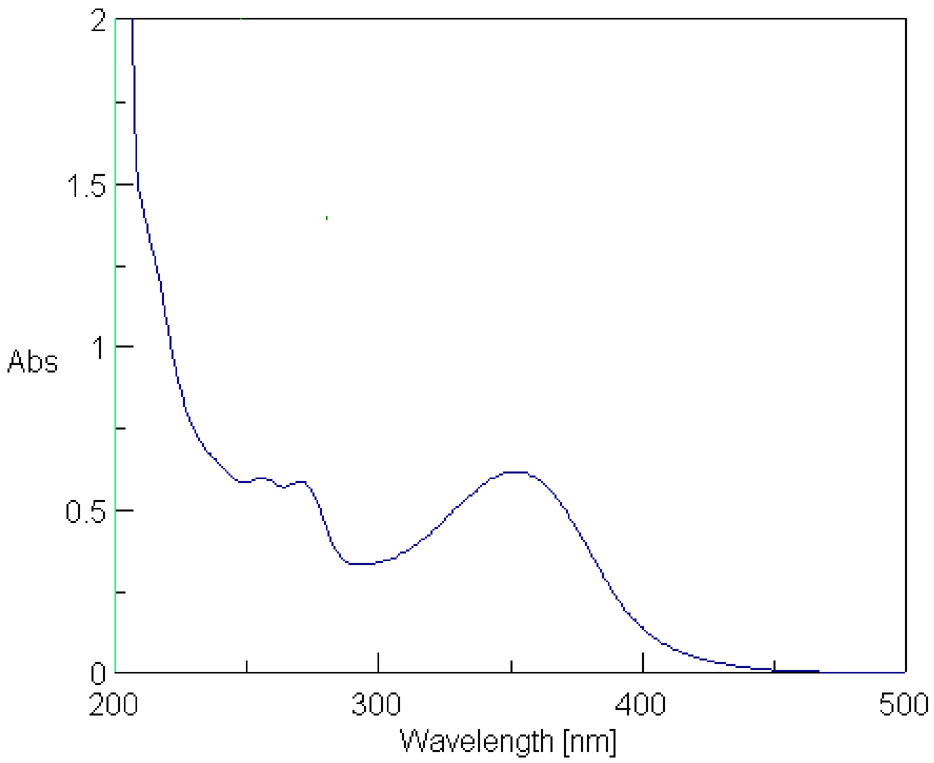

Figure S4. UV spectrum of jaceidin in MeOH.

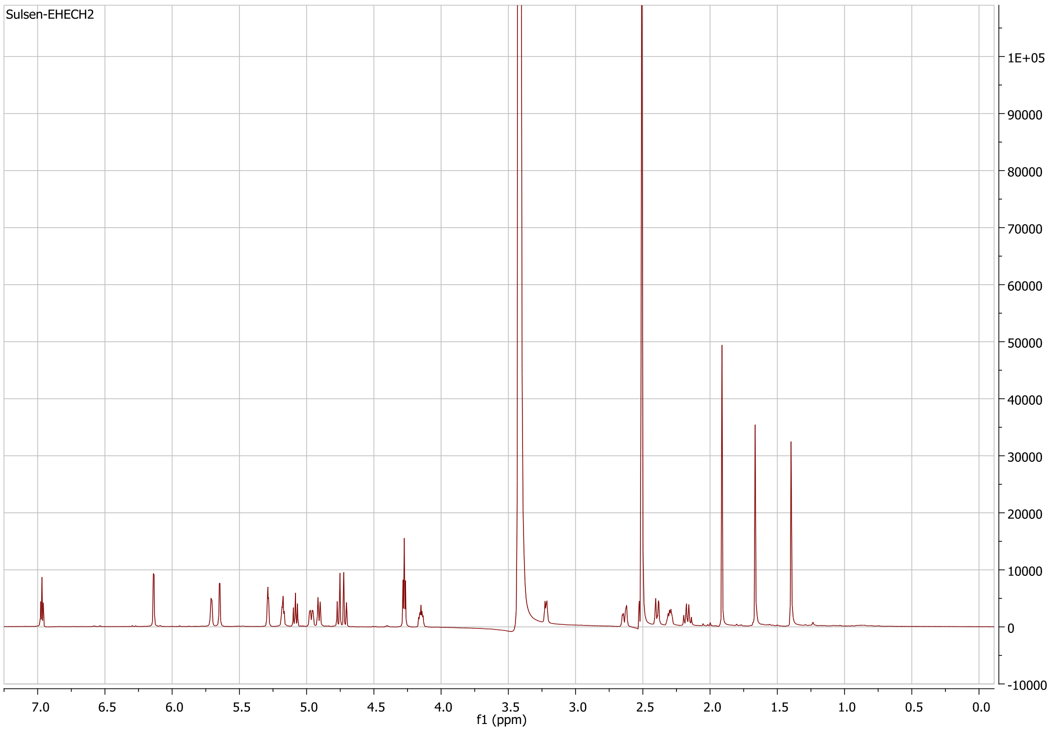

Figure S5. ^{1}H-NMR spectrum of santhemoidin C in DMSO-d_6.

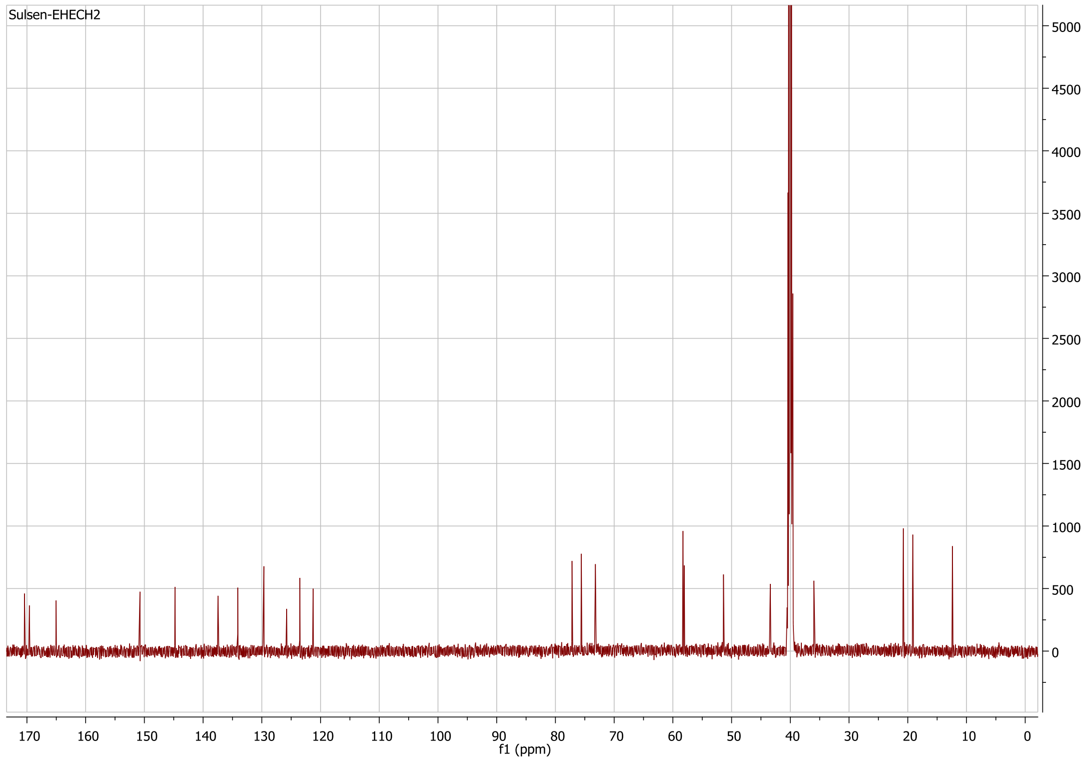

Figure S6. ^{13}C-NMR spectrum of santhemoidin C in DMSO-d_6.

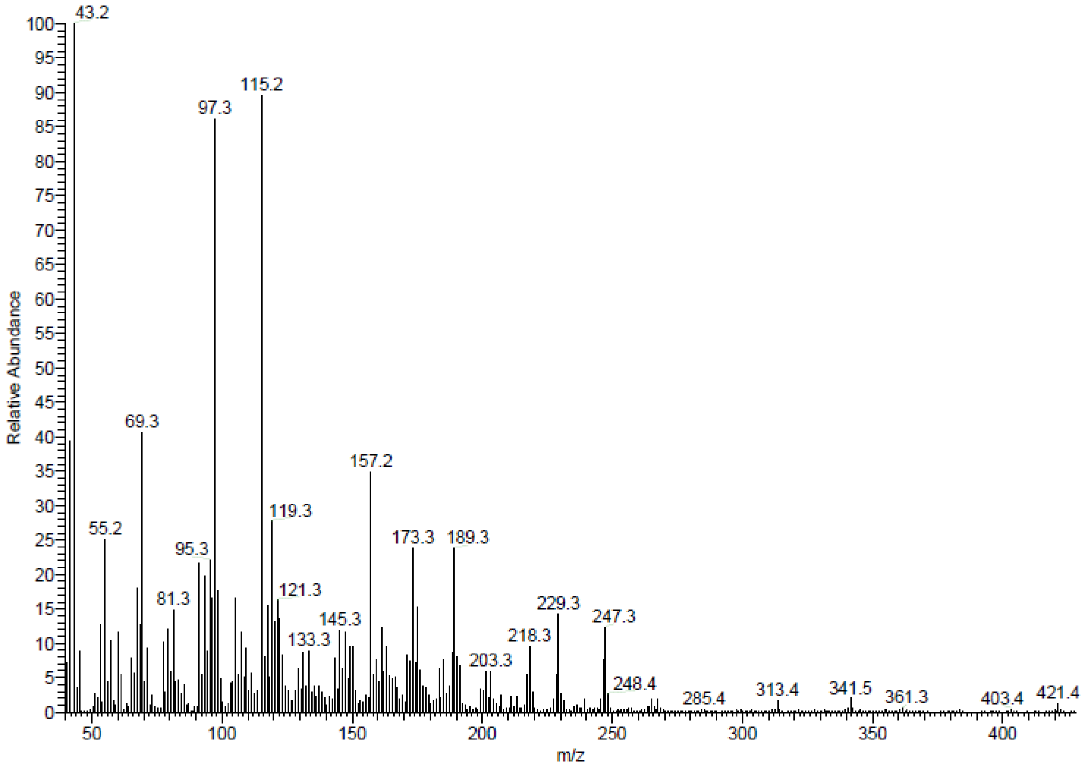

Figure S7. EI-MS spectrum of santhemoidin C (70 eV).

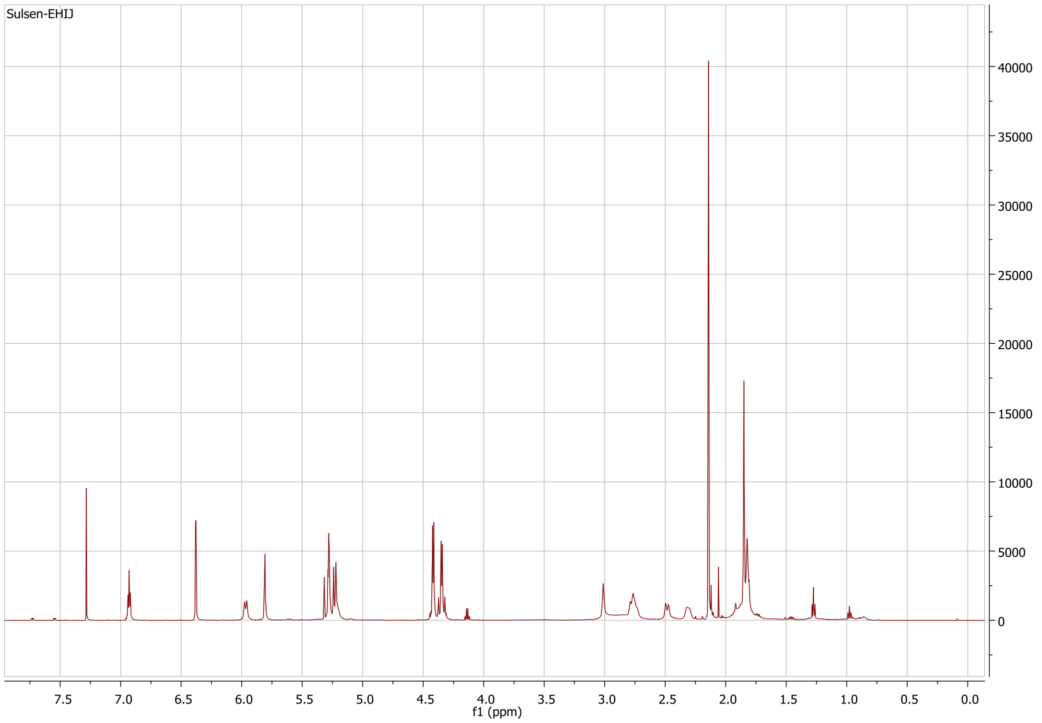

Figure S8. ^{1}H-NMR spectrum of eucannabinolide in $CDCl_3$.

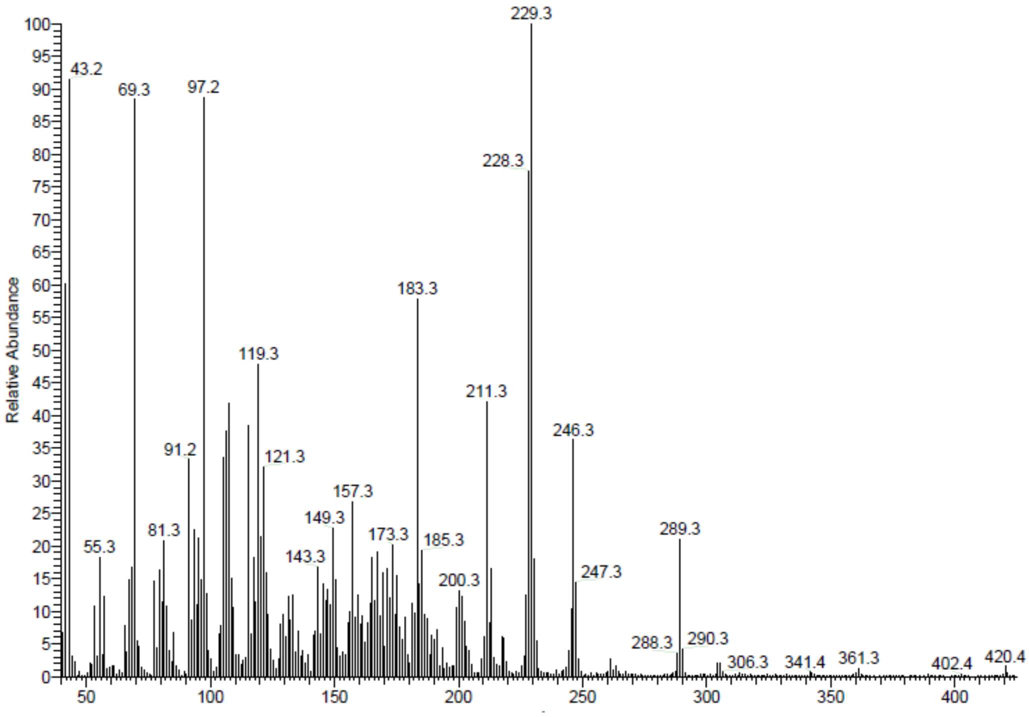

Figure S9. EI-MS spectrum of eucannabinolide (70 eV).
